# Inflammation and Necrosis Syndrome in Young Piglets—A Longitudinal Study

**DOI:** 10.3390/vetsci12080752

**Published:** 2025-08-13

**Authors:** Sabrina Becker, Katrin Hindenlang, Josef Kuehling, Mirjam Lechner, Gerald Reiner

**Affiliations:** 1 Justus-Liebig-University Giessen, Department of Veterinary Clinical Sciences, Clinic for Swine—Herd Health Management and Molecular Diagnostics, Frankfurter Strasse 112, 35392 Giessen, Germany; eisenhofer.katrin@googlemail.com (K.H.); josef-kuehling@web.de (J.K.);; 2 UEG Hohenlohe, Am Wasen 20, 91567 Herrieden, Germany

**Keywords:** animal welfare, swine

## Abstract

Swine Inflammation and Necrosis Syndrome (SINS) is a simple and non-invasive animal-based health and welfare indicator that combines the clinical observation of bristle loss, swelling, redness, exudation, necrosis and haemorrhage in various parts of the body. It provides a point-of-care measure with direct intervention capability. Several studies from different countries demonstrate its considerable prevalence, particularly among newborn, suckling and weaned piglets. The syndrome has been demonstrated to be endogenous, as evidenced by clinical, pathohistological, clinical chemical, metabolomic, transcriptomic and genomic analysis. It has been established that the first and fourth weeks of life represent suitable time points for examination. However, longitudinal follow-up of individual animals has hitherto been lacking. In order to address this issue, a total of 1080 complete SINS examinations were conducted on 59 piglets at days 1 to 14, 19, 22, 26 and 41 of life. The findings substantiate the bimodal progression and evince a robust correlation between signs in disparate anatomical regions, including body temperature. Two peaks with significantly increased SINS signs were observed, the first around the fourth day of life and the second around day 26. The majority of indications of SINS in the second peak manifested prior to the initiation of the weaning process. The development of SINS signs in the piglets as a group followed a clear pattern. However, it was not feasible to predict the subsequent course of SINS based on individual animals. It is recommended that SINS, as an animal-based health and welfare indicator, be screened on days three to four and/or in the fourth week of life. It is imperative that the day of life is specified with the greatest possible precision, given the propensity for considerable deviations to occur within a time frame of one to three days, especially during the initial week of life. The implementation of these findings has the potential to make a decisive contribution to improving inventory herd analyses and studies on SINS, thereby improving the welfare and health of piglets.

## Simple Summary

Piglets can sometimes have skin alterations and lesions on their ears, tails or claws, even in the first days of life. All these changes together are called Swine Inflammation and Necrosis Syndrome (SINS). Until now, it was unclear how these characteristics develop over time in individual animals. In this study, we looked at piglets from when they were born until they were 41 days old. We wanted to know when and how the SINS characteristics appear and change over time. The most cases of the disease were seen in the first and fourth weeks of life. Things got better in between. Some piglets showed the most symptoms at both times, while others only showed symptoms in the first or fourth week of life. The results help us to better understand SINS. The findings from individual animals in the first week can only be used to draw limited conclusions about their condition in the fourth week. These findings will help to improve the analysis of pig herds and studies on SINS. This will help to improve the welfare and health of piglets.

## 1. Introduction

The screening of inflammation and necrosis is utilised as an indicator of the health and welfare of newborn, suckling and weaned piglets [1,2,3]. The process under discussion involves the combination of different characteristics in different parts of the body, thereby forming Swine Inflammation and Necrosis Syndrome (SINS) [4]. This screening system has been developed to provide animal keepers and veterinarians with a point-of-care method, thereby enabling them to intervene directly when serious signs can still be prevented [1]. The method is distinguished by its specificity, in that clinical findings can be obtained solely by clinical observation during routine handling of the piglets, without the need for invasive, costly or time-consuming procedures. The collection of specific binary data on the presence or absence of bristle loss, swelling, redness, rhagades, exudation, necrotic tissue and spontaneous bleeding is of particular importance [4]. The presence of bite marks can be excluded through the identification of counterbite marks or the evaluation of bite wounds. In order to achieve greater precision, it is possible to calculate the body part scores and subsequently derive the SINS score from the binary data. This approach serves to mitigate the challenges associated with the subjective collection of ordinal scores. The tail is typically divided into a base and a tip, with the ears, teats, coronary bands and heels also being included. In some cases, the navel, vulva, or face may also be included [5,6,7]. Consequently, minor discrepancies may emerge among studies due to alterations in the scoring matrix. However, these variations do not impact the fundamental principle or the absolute comparability within the same system. Practical experience suggests that tail lesions are not only caused by tail biting, but also by inflammation and necrosis, which can occur independently of contact with other pigs. Furthermore, these symptoms affect not only the tail, but also the ears, heels, soles, coronary bands, teats, navel, vulva and face. Studies from Germany [2,7,8], France [3] and the Netherlands [1] have demonstrated the presence of SINS in piglets of various ages. On average, 30–40% of animals were affected, with the most severe symptoms experienced by weaners, followed by suckling piglets. SINS signs were also detected in finishers, which were associated with the sow’s condition and husbandry practices from weaning to finishing [8]. Due to the simultaneous occurrence of inflammation in different body parts and the detection of SINS signs in newborn piglets within two hours of birth, the hypothesis of an endogenous primary event was formed [5]. Over 80% of newborn piglets showed alterations to the tail base, claw wall and heels. In 65–87% of animals, the coronary bands, teats, face and ears were affected. With an average of six out of nine body parts affected, none of the piglets were completely free of pathological signs. Histopathological characterisation of the piglets revealed an intact epidermis but numerous granulocytes, macrophages and lymphocytes in the subepithelial connective tissue. Such an accumulation is not expected until 4–7 days after an inflammatory trigger [9]. In suckling, weanling and finishing piglets with SINS, signs of inflammation in areas with clinical alterations were also evident, including vasculitis, intima proliferation and thrombotic changes [8]. Systemic inflammation and haemostasis disorders were evident in clinical chemical analyses of serum from piglets [10]. Higher degrees of clinical SINS were associated with increased numbers of monocytes and neutrophils. Blood coagulation was altered in weaners, and thrombocytopenia was observed in fatteners. Additionally, acute-phase proteins, especially C-reactive protein and fibrinogen, were found to be elevated in the serum. Serum liver enzymes were slightly altered. Overall, aspartate transaminase levels exceeded physiological limits and increased in parallel with SINS scores in fattening pigs. The generalised inflammatory nature of SINS was also demonstrated by comparing the liver gene expression of piglets with and without obvious signs of SINS [11]. Several pro-inflammatory, acute-phase and stress-response genes were found in piglets with SINS. The transcriptomic findings were supported by metabolome analysis results. Deviations in metabolic metabolites, particularly changes in lipid metabolism in piglets with pronounced SINS symptoms, supported the transcriptomic abnormalities and indicated liver stress induced by metabolism. These results provided the first evidence of an inflammatory process induced in the livers of piglets with SINS, accompanied by deranged lipid metabolism. Gerhards et al. [12] further characterised this inflammatory aspect of SINS, emphasising the importance of inflammatory and defence genes such as CRP, as well as other candidate genes including S100A12, GYPA and LIPK. These studies prove that SINS is not merely a local skin condition, but also involves hepatic inflammation and metabolic dysregulation, as demonstrated by transcriptomic and metabolomic analyses.

It has been assumed that SINS scores in piglets are not constant over time; rather, they have been observed from days 2 to 8 in some studies [1], but mainly on days 3 to 4 and approximately 10 days after weaning. This assumption was based on a series of practical observations. Consequently, studies have been conducted at these specific time points [4,5,6,8]. However, there is a paucity of longitudinal studies in individuals that could confirm these assumptions and recommendations. Consequently, the extent to which the SINS dynamics observed to date are based on comparisons between different individuals of suckling piglets and weaners remains unclear. This could result in inconsistency when comparing studies and interpreting their results.

The objective of the present study was, thus, to analyse the dynamics and significance of SINS characteristics in individual piglets. The following hypotheses were formulated:

1. Dynamics of characteristics: The temporal dynamics of SINS characteristics in different body regions exhibit distinct patterns during the suckling period, both at the group level and at the individual level.

2. Time-defined peaks: SINS scores attain characteristic zeniths within designated time windows, with the timing and severity of these maximum values varying depending on the characteristic and the individual.

3. Sequence of severity levels: The development of SINS characteristic severity levels follows a typical chronological sequence that is recognisable in the majority of piglets.

4. Correlation with body temperature: There is a substantial correlation between SINS scores and body temperature.

5. Predictive value for the weaning phase: The severity of SINS characteristics in individual suckling piglets is correlated with their severity after weaning.

## 2. Materials and Methods

### 2.1. Study Design

The present study was conducted in accordance with the Declaration of Helsinki. The experiment comprised non-invasive examinations only. The sole procedure entailed the photographic and visual collection of clinical scoring results. Consequently, the experiment was not classified as animal testing by the relevant authorities (the Giessen Regional Council, listed under file number 54/19/C/20/15/H/02/GI/18/15/KTV/3/2019).

The 59 piglets were derived from four litters that were randomly selected from a group of eight sows (every second sow according to sow number) in the farrowing group on the day of birth. The study commenced on 1 November 2019 with the first two litters and continued on 13 February 2020 with the next two litters, concluding on 16 April 2020.

The litters were utilised in their totality, with 13, 14, 15 and 17 piglets, respectively. The sows were a cross between the German Landrace and Large White breeds. The insemination of the sows was conducted using semen from a number of Pietrain boars. It is important to note that the present pilot study was not intended to collect representative data for the diverse breeds under various husbandry conditions.

### 2.2. Housing, Feeding and Zootechnical Measures in Sows

The sows and their piglets were accommodated in farrowing pens measuring 5.3 m^2^, with plastic flooring. The sows were confined within a farrowing crate, which was constructed from cast iron and contained a lying area. The sows were fed mash feed three times a day using a Spotmix system (Schauer Agrotronic GmbH, Prambachkirchen, Austria). The feed composition for lactating sows was 16% crude protein, 5.0% crude fibre, 3.3% crude fat, 0.8% calcium, 0.5% phosphorus, 0.2% sodium, 0.9% lysine and 0.3% methionine, with a total energy content of 12.7 MJ of metabolizable energy (ME)/kg. Water was made available to the subjects via drinking troughs. On the 14th day of lactation, the sows were vaccinated against erysipelas and parvovirus (Porcilis^®^ Ery + Parvo, MSD Animal Health, Munich, Germany). Two weeks prior to farrowing, the sows were vaccinated against Clostridium perfringens (Clostriporc^®^ A, IDT Biologika, Dessau-Rosslau, Germany).

### 2.3. Housing, Feeding and Zootechnical Measures in Piglets

The suckling piglets were provided with a piglet nest that was equipped with zone heating and a heat lamp. On the day of birth, the standard zootechnical measures implemented included the grinding of the canines’ tips, with the objective of minimising injury to the animals and the udder. Moreover, the animals were subjected to ear-tagging for the purpose of identification, and oral iron supplementation was administered.

On the fourth day of life, the male piglets underwent castration under anaesthesia induced by an injection of ketamine (Ursotamin, Serumwerke Bernburg, Germany) and azaperone (Stresnil, Elanco, Germany) at a ratio of 4:1 and a dosage of 1 mL per 10 kg body weight, administered intramuscularly. The piglets were vaccinated with Porcilis^®^ PCV/M. Hyo (MSD, Munich, Germany) five days prior to weaning, on day 28 of life.

During the rearing phase, the piglets were housed in pens measuring 5.3 m^2^, with plastic flooring. The subjects were provided with unlimited access to food, and water was supplied via nipple drinkers. The weaning feed, designated as 1, was utilised for piglets with a live weight of 8–12 kg, while weaning feed 2 was employed for those with a live weight of 12–20 kg.

The formulation of weaning feed 1 was as follows: 16.6% crude protein, 3.5% crude fibre, 4.7% crude fat, 0.8% calcium, 0.5% phosphorus, 0.2% sodium, 1.3% lysine and 0.4% methionine. The total energy content of the feed was determined to be 13.5 MJ ME/kg. Following the transition to feed 2, the composition was ascertained to be 18% crude protein, 3.6% crude fibre, 2.6% crude fat, 0.8% calcium, 0.5% phosphorus, 0.2% sodium, 1.2% lysine and 0.4% methionine, with a total energy content of 13 MJ ME/kg.

### 2.4. Clinical Scoring of SINS Characteristics

Each piglet was examined on days 1–14, 19, 22, 26 and 41 of life. Due to temporal constraints and with a view to reducing the burden on the animals, clinical signs were documented by means of a digital camera. The Canon EOS 1200D model, equipped with an EF-S 18-55 mm lens, was utilised for the photographic documentation of the subject (Canon, Krefeld, Germany). The image sensor has a resolution of 18 megapixels. The camera is equipped with a 3-inch LCD monitor and a built-in flash mechanism. The images were stored on an SD memory card, a device which facilitated their transfer to a computer. Prior to the procedure, it was necessary to clean the piglets’ claws. The cleaning process was conducted using a mixture of lukewarm water and soap, with a damp cloth being employed for the task. A thorough evaluation of the images was conducted by employing the Windows Media Player software (Version 12, Microsoft GmbH, Munich, Germany).

The tail (including the base and tip), ears, teats, claws (with coronary bands), heels and hind limbs were photographed individually. Subsequently, the photographs were subjected to a blind evaluation by a single assessor in a quiet environment. The presence (1) or absence (0) of different lesions on different parts of the body was noted in a binary manner. A thorough examination of the tail base was conducted to ascertain the presence of any indications of loss of bristles, swelling, redness, exudation and necrosis. A comprehensive evaluation was conducted on the remaining portion of the tail, encompassing the distal tip, to ascertain the presence or absence of bristles, as well as the existence of any palpable swelling, scabs, rhagades, exudation, necrosis, ring constriction and bleeding. The ears were examined for indications of bristle loss, venous congestion and necrosis. The teats were subjected to a thorough examination for the presence of signs of venous congestion, inflammation, redness, scabs and necrosis. The claws were screened separately on the anterior and posterior limbs. The presence of signs was documented as 1 if they were observed on at least one of the eight claws. The claw wall was examined for indications of swelling. The coronary bands were subjected to a thorough examination for any signs of swelling, redness, exudation or clefts. The heels were examined for signs of swelling, redness and tearing. In addition, the hind limb was subjected to screening for signs of venous congestion. All screening was performed in accordance with [4].

### 2.5. Measurement of Body Temperature

The body temperature was measured at three locations: the base and tip of the tail, the ears and the teats. In order to achieve this objective, an infrared camera manufactured by FLIR Systems GmbH (Frankfurt/Main, Germany) was employed. The T540 model is a handheld infrared camera with a field of view of 42° × 32°, a minimum focus distance of 0.15 m and a resolution of 464 × 348 pixels. The device is equipped with an integrated digital camera in addition to the infrared camera. The detector under consideration is a focal plane array that incorporates an uncooled microbolometer. The camera is equipped with a 4-inch swivel monitor and offers both manual and automatic focusing capabilities. The geometric resolution of the system is 1.66 mrad/pixel, operating at a frame rate of 30 Hz and with an accuracy of ±2 °C within the temperature range of measurement objects ranging from −20 °C to +100 °C. The configuration of object parameters is facilitated within the camera software (Version 5.13), and the images are stored on SD memory cards, with the option of transfer to a computer. The evaluation of the infrared thermographic images was conducted utilising FLIR Tools (FLIR Systems GmbH, Frankfurt/Main, Germany).

The object parameters, the emissivity of pig skin (=0.98) and the distance between camera and object (=1 m), were set to constant values, while the parameters relative humidity and room temperature were set to 50% and 22 °C in the software, as recommended by the manufacturer.

The reflected temperature was determined by taking an image with aluminium foil adhered to cardboard. The mean temperature of the image was determined by employing a large measuring rectangle in the FLIR Tool. In order to execute this process, it was necessary to set the emissivity to 1 and the distance to 0 m. The reflected temperature, as determined by this method, was then entered into the FLIR Tool for the subsequent images.

The emissivity was set to a constant 0.98, in accordance with the specifications outlined in [13]. The temperature range setting demonstrated no significant impact on the measured values. However, it facilitated thermal optimisation, thereby elucidating thermal patterns within the range of 20 °C to 40 °C.

The infrared images were invariably captured concurrently during the morning hours, between 9:00 and 12:00.

For each ear, five measurement points were placed, starting at the base and moving upward to the tip. The mean of the 10 values was then employed to calculate the ear temperature. The temperature of the tail was measured along the midline, from the distal tip to the base. Subsequent evaluation was conducted by calculating the difference between the temperature of the distal 10% of the tail (i.e., the tail) and the proximal 10% of the tail (i.e., the tail base). The magnitude of temperature differences exhibited increased in proportion to the extent of cooling from the tail base to the tail tip. The mean temperature of the seven pairs of front teats was calculated in order to determine the teat temperature, due to the incomplete availability of data for teats located further posteriorly on both sides.

### 2.6. Statistical Analysis

All data were collected and prepared in Microsoft Excel (Microsoft, Munich, Germany) prior to being analysed using IBM SPSS Statistics version 27 (IBM, Munich, Germany). The prevalence of the examined signs in the various body parts was calculated based on binary findings. Furthermore, the total sum of all binary data pertaining to a specific body part was calculated to derive the respective body part score (e.g., tail score). Subsequently, the scores for each body part were aggregated to derive the SINS score. A factor analysis with rotation using the varimax method was used for the principal component analysis. In order to ensure absolute comparability between individual body part scores, the scores were subjected to a Z-transformation and subsequently plotted as a function of age. A general linear mixed model was utilised to calculate the significance between days of life. The piglet was designated as the subject ID. The day of life was utilised as the repeated measure. Sex was considered a fixed effect, whilst litter was considered to be a random effect. Spearman’s rho correlations were utilised to analyse the data, a method that is not contingent upon the distribution of the data. The times of first, mean and last appearance, as well as the earliest mean and last time of visibility of signs of SINS, were calculated for each individual piglet for descriptive presentation. The effect of the sign, independent of the body part, was subjected to calculation using a one-factorial analysis of variance. The presence of SINS peaks was identified for each individual, provided that these peaks were higher than 1.5 standard deviations of the mean. It was conceivable for an individual to exhibit multiple peaks. The repeatability of the results over a period of days of life was demonstrated using the coefficients of determination for the correlations between values on each pair of consecutive days. Statistically significant differences between values were defined as those with *p*-values less than 0.05.

## 3. Results

### 3.1. Prevalence of SINS Characteristics in the Piglets and Body Temperature

The majority of piglets exhibited signs of bristle loss (100%) and swelling at the base of the tail tip (83.1%) from the first day of life (see Table 1). By day 41, the respective prevalence had decreased to 38.5% and 69.2%, respectively.

As demonstrated in Table 1, a greater number of piglets exhibited more pronounced clinical signs, with the most prevalent signs including redness at the base of the tail and exudation, primarily observed during the initial week of life. Necrosis was not observed. Furthermore, between days 5 and 22, the tail was observed to be swollen in 18–22% of piglets. Scabs, rhagades, exudation and spontaneous haemorrhages were observed in only a few animals, peaking in the first days of life and from the third week onwards. The study revealed that necrosis and ring constriction were not observed in the studied group (Table 1). Exudation at the base of the tail invariably ensued subsequent to the occurrence of swelling and redness.

A more or less severe loss of bristles on the ears was observed in almost all of the examined piglets and could, therefore, not be used for further differentiation. Furthermore, congestion of the ear veins was observed in the majority of the piglets. Necrosis of the ears did not occur until the third week of life (Table 2).

As many as 30–40% of the piglets presented with signs of swelling and erythema from the first day of life. Furthermore, the onset of teat necrosis was observed as early as day 1, as detailed in Table 2. Moreover, the prevalence of teat necrosis among the piglets escalated to over 20% by day 4.

The coronary bands demonstrated two phases of swelling, with a minimum observed between days 13 and 19. Subsequent to this, exudative inflammation ensued, with clefts manifesting approximately one week later, initially on the forelimbs and subsequently on the hindlimbs (see Table 3 for details).

The heels exhibited a marked redness, more pronounced in the forelimbs than the hindlimbs. As demonstrated in Table 3, the initial swelling decreased by the second week of life, but then increased again from the third week onwards. Heel lacerations manifested with a delay of approximately one week. As early as the second week of life, signs of claw swelling became increasingly apparent. From three weeks of age, congestion of the hind limb veins was evident (Table 3).

An increase in temperature was observed from day 1 to days 4 and 5 at the base of the tail, at the ears and the teats. The increase in temperature at the tail tip persisted until the seventh day. Subsequently, a decline in temperature was observed (Table 4). The reduction in temperature from the tail base to the tail tip exhibited two maximum levels around days 3 and 4, and around day 26.

### 3.2. Principal Components of SINS Characteristics

**Figure 1:**
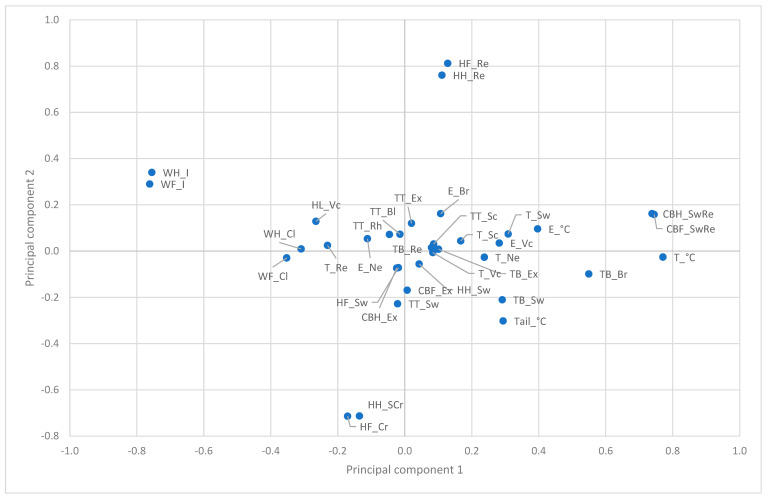
Principal component analysis of SINS characteristics. The first and second components, C1 and C2, are shown. Letters before the underscore: TB: tail base; TT: tail tip; E: ear; T: teats; CBF: coronary bands, front limb; CBH: coronary bands, hind limb; HF: heels, front limb; HH: heels, hind limb; WF: claw wall, front limb; WH: claw wall, hind limb; Letters behind the underscore: Bl: bleeding; Br: no bristles; Cl: cleft; Cr: crack; Ex: exudation; I: swelling/bleeding, cracks in claw wall; Ne: necrosis; Re: redness; Rh: rhaghades; Sc: scabs; Sw: swelling; Vc: venous congestion; °C: temperature.

A thorough analysis of the principal components of the SINS characteristics, independent of the day of life, revealed 14 significant components, collectively accounting for 64% of the variance across all characteristics. The initial two principal components accounted for 17% of the variance (components C1: 11% and C2: 6%). The analysis confirmed the close relationship between characteristics of different parts of the body (Figure 1). Exudation of coronary bands was also closely associated with the other SINS characteristics. Conversely, findings on the heels, claw wall and swelling/redness on the coronary bands exhibited only a weak association.

### 3.3. Tail and Tail Base

In order to facilitate the comparison of the individual parameters in relation to their respective courses over the course of the study, the Z-transformed values were utilised below. This resulted in a mean of 0 and a standard deviation of 1 for all the characteristics. The signs that were observed in almost all piglets, namely the absence of bristles, swelling at the base of the tail and tail tip swelling, were not listed again explicitly.

**Figure 2:**
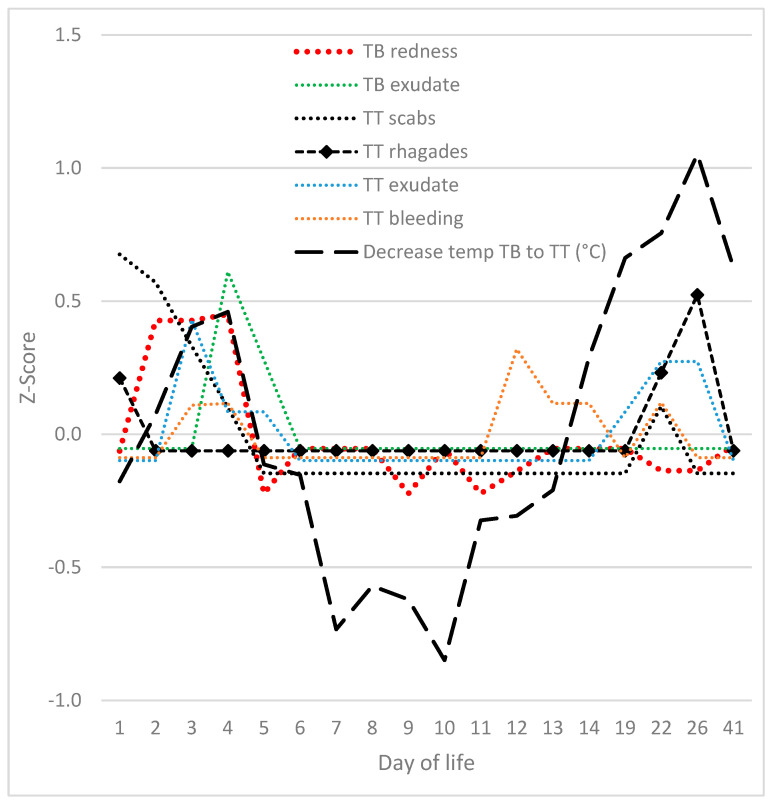
Relative development of signs (Z-scores) on the tail from the first to the 41st day of life. TB: tail base; TT: tail tip and rest of the tail.

The progression of visible clinical signs on the tail exhibited two distinct peaks (Figure 2). The initial occurrence manifested within the first five days of life. The manifestation of erythema at the base of the tail reached its zenith on days 2–4. Levels were found to be significantly higher than on any other day. Exudation at the tail base reached its maximum on day 4 (exceeding levels on any other day, with the exception of day 5). The presence of scabs on the tail reached its zenith on days 1 and 2, at which points the levels were significantly higher than at any other point, with the exception of day 3. The presence of rhagades was observed at the tail on day 1, reaching a peak on day 26 (exhibiting significantly higher values than on days 1 and 22). Exudation at the tail was observed to be at its peak on day 3 (significantly exceeding the levels recorded on days 1, 2, 6–14 and 41) and on day 22 (also significantly exceeding the levels recorded on days 1, 2, 6–14 and 41). Tail bleeding was observed in a few piglets on days 3 and 4 (statistically not significant) and on day 12 (significantly different from days 1, 2, 5–11 and 19), as well as on day 22 (statistically not significant). The decrease in temperature from the tail base to the tail tip followed a similar pattern, with peaks observed on days 3 and 4, and on days 19 to 41. The decline in temperature from the tail base to the tail tip was found to be considerably more marked than that documented on days 1 and 5–13. With the exception of the haemorrhaging observed on day 12, no abnormalities were detected between days 6 and 19 of life.

**Figure 3:**
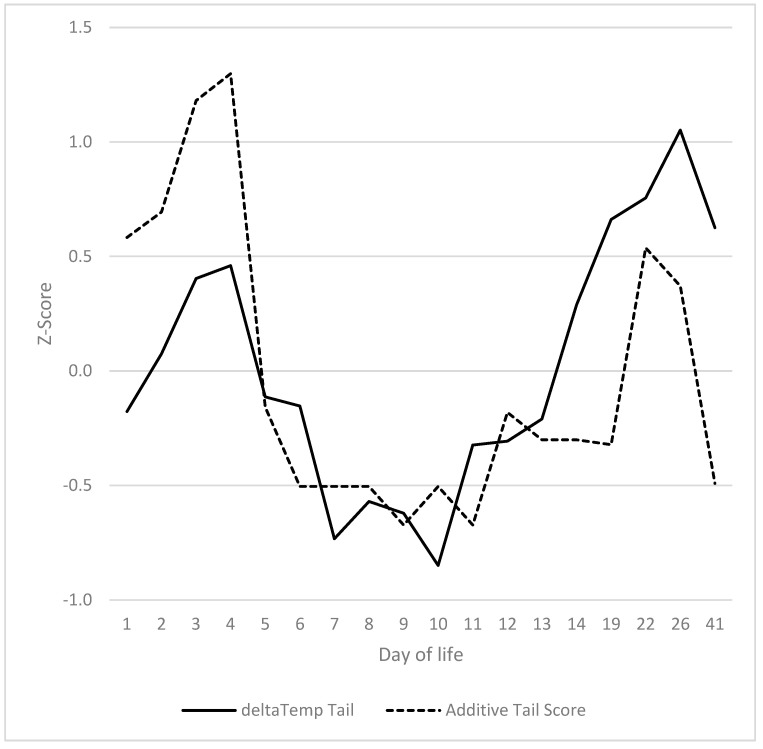
Overlap of additive tail score (including all visible parameters from Figure 2) with the decrease in temperature from tail base to tail tip.

In summary, a high correlation was observed between the progression of clinical signs at the base of the tail or on the tail and the decline in temperature from the base of the tail to the tip (Figure 3). The correlation between the two was found to be r = 0.528 (*p* = 0.007). The only differences observed were in the relative severity of the signs when compared to the decline in temperature. The temperature peaks and additive tail scores were found to be significantly higher during days 6 to 14 (*p* < 0.05).

Subsequent alterations in the tail tip occurred with a delay of approximately four days, following alterations in the tail base. Changes in the tail tip occurred subsequent to the attainment of maximum levels in the tail base.

### 3.4. Ears

**Figure 4:**
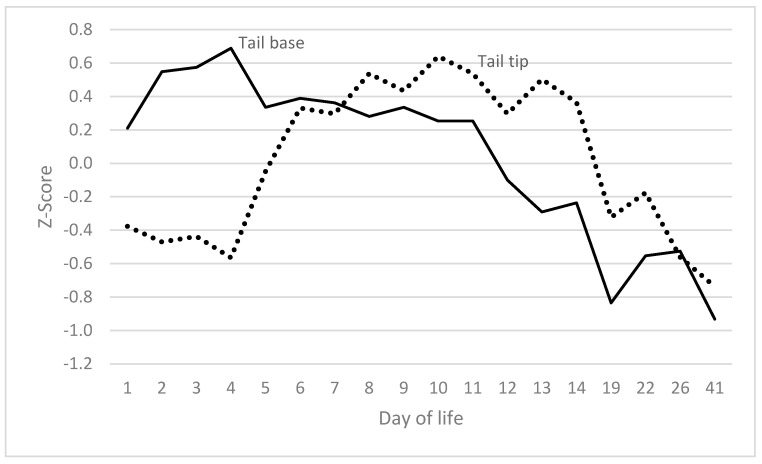
Changes in tail tip follow changes in tail base with a time lag of around 4 days.

A significant correlation was identified between ear score, ear vein score and ear temperature (Figure 4). It was determined that the absence of bristles on the ears of virtually all piglets rendered this feature moot for the purposes of the analysis. The correlations of ear score to ear vein score, ear score to ear temperature and ear temperature to ear vein score were 0.661 (*p* = 0.002), 0.695 (*p* < 0.001) and 0.461 (*p* = 0.47), respectively. The ear score was recorded subsequent to the ear vein score, with a delay of approximately 24 h. The aforementioned signs were accompanied by a parallel course of ear temperatures. The ear score on days 4 and 7 was found to be significantly higher than on days 11 to 22, and significantly lower than on day 41. The ear score during the second peak (day 41) was significantly higher than on any other day of life. The ear vein score demonstrated a congruent trajectory with the ear score. Statistically, the ear vein score was found to be significantly elevated on days 5 and 41 in comparison to days 14 and 22. A significant increase in ear temperature was observed on days 5 and 41 (corresponding to the first and second peak, respectively) in comparison to days 8–26.

**Figure 5:**
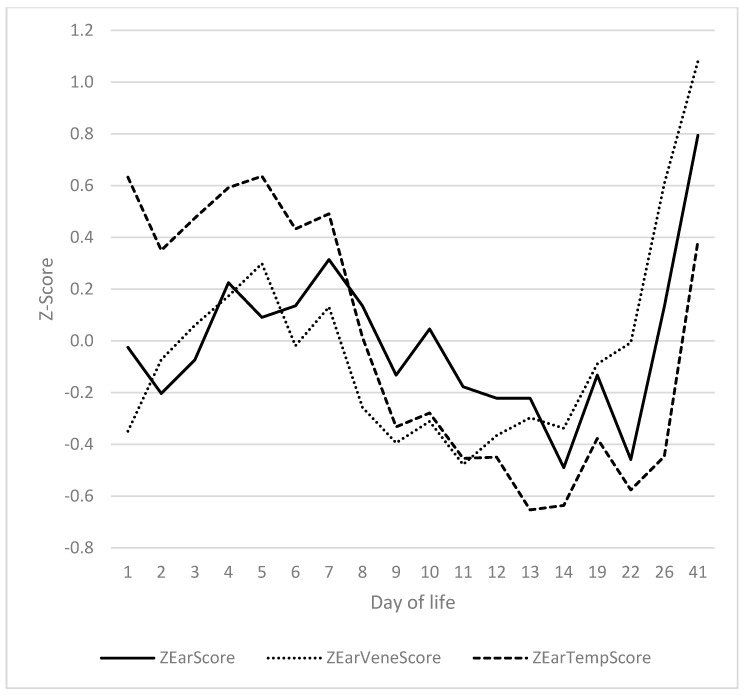
Ear score, ear vein score and ear temperature from day 1 to day 41.

The courses of the ears exhibited a close correspondence to those of the tail, with a slight time lag. A significant correlation of 0.582 (*p* = 0.009) was identified between the ear vein score and the decrease in tail temperature from base to tip (Figure 5).

### 3.5. Teats

**Figure 6:**
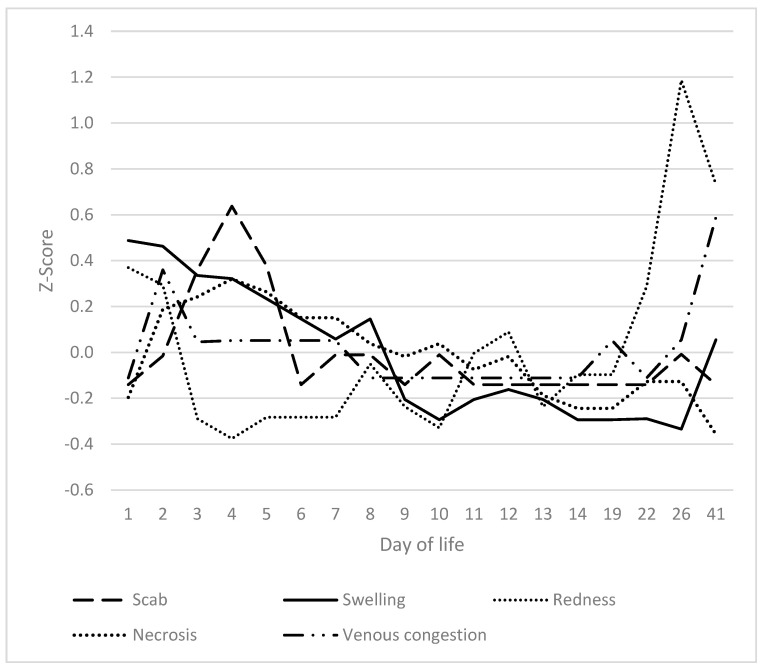
SINS signs at the teats by day of life.

The teat scores demonstrated a comparable trend (see Figure 6), manifesting two peaks: the first occurring during the initial week, and the second commencing on day 22. On the first day of life, the teats exhibited signs of increased swelling and redness. As indicated by the presence of signs of venous congestion, including scab formation, and the initial occurrence of teat necrosis, the second day was marked by the onset of these symptoms. Scabs and necrotic lesions were evident during the first week of life. Subsequent to this, the signs underwent a period of abatement. As of the third week of life, there was a renewed increase, particularly with regard to teat redness and venous congestion. The presence of scabs at the teats reached its zenith during days 3 to 5, at which time levels were significantly higher than on any other day. The highest values for teat swelling were recorded during the first two days of life (a significant difference from days 7 to 41). The preliminary observations indicated that the teats displayed a degree of erythema that became apparent during the initial 48 h of life. This finding was found to be significantly different from all other days, with a statistical significance of *p* < 0.05. Furthermore, it was observed that the teats continued to exhibit redness on days 12 and 26, with a statistical significance of *p* < 0.05, as compared to all other days. The incidence of teat necrosis reached its peak on day 4, which was significantly higher than on days 1 and 13–41. The occurrence of venous congestion of the teats was identified, exhibiting two peaks: on day 2 (exhibiting a significantly higher level than on days 1, 8–14, and 22) and on day 41 (significantly higher than on all other days, but day 2).

The investigation revealed no substantial impact of gender on any of the parameters, with the exception of the teats. A statistically significant difference was observed in the teat score between male and female subjects on days 1 to 9 and 14 (*p* < 0.001). A detailed analysis revealed that there were no statistically significant differences between sexes with regard to the prevalence of teat scabs or venous congestion. A marked increase in the incidence and severity of swelling of the teats was observed in female piglets in comparison to males on a daily basis. As demonstrated in Figure 2, the manifestation of redness of the teats was more pronounced in females than in males on days 22 and 26. The incidence of teat necrosis was found to be significantly higher in female subjects than in males on days 1, 4, 11 and 12.

**Figure 7:**
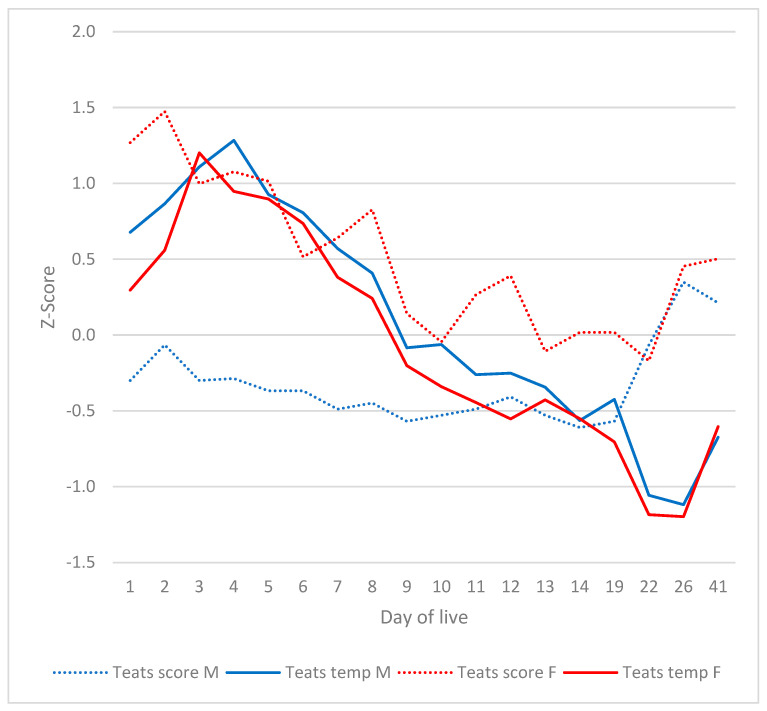
Teats score and teats average temperature by day of life and gender. M: male piglets; F: female piglets; temp: average temperature.

The occurrence of marked deviations in clinical signs and teat temperature was observed to coincide. The investigation revealed that the teat temperatures of male and female piglets were indistinguishable. At the time of the initial SINS peak, teat temperatures were considerably higher than those observed after day 8. As illustrated in Figure 7, at the time of the second SINS peak, the teat temperature was found to be significantly lower than it had been prior to day 22. The investigation revealed no statistically significant difference in teat temperature between male and female piglets (Figure 7). The mean teat temperature was found to be significantly higher on day 4 than on day 1 and on days 7–41 (Figure 7). A significant decrease in teat temperature was observed on days 22 and 26 in comparison to days 1–19 and 41. In the male piglets of the study, a significant increase in teat temperature was observed on days 4 and 5 in comparison to days 1 and 5–41, respectively. On days 22 and 26, teat temperatures were significantly lower than on days 1–19 and day 41. A meticulous examination revealed no substantial disparities between the front and rear teat pairs, nor between the right and left sides.

### 3.6. Heels, Coronary Bands and Claws

**Figure 8:**
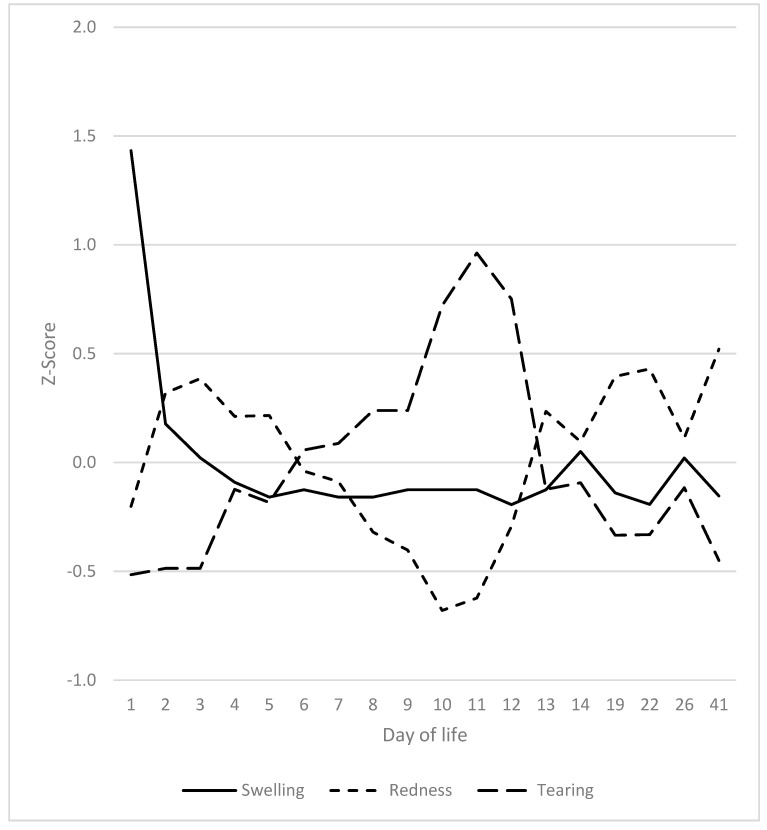
Clinical signs at the heels by day of life.

Excessive swelling of the heels was observed from the first day of life and decreased to a basal level by day 3 to 4 (Figure 8). The values recorded on the first day were significantly higher than on any other day. As the inflammation subsided, the erythema of the heels intensified, reaching its zenith on the third day. The severity of redness exhibited a downward trend during the initial phase, spanning from days 7 to 12, followed by an uptick beginning on day 13. By day 10, the extent of redness had significantly diminished in comparison to days 1 to 8 and 12 to 41. The third sign that increased was tearing of the heels, with a peak between days 4 and 14. The maximum observed on day 10 exhibited a significant deviation from the values recorded on days 1 to 9 and 13 to 41. The redness of the heels followed the general two-peak course of SINS.

**Figure 9:**
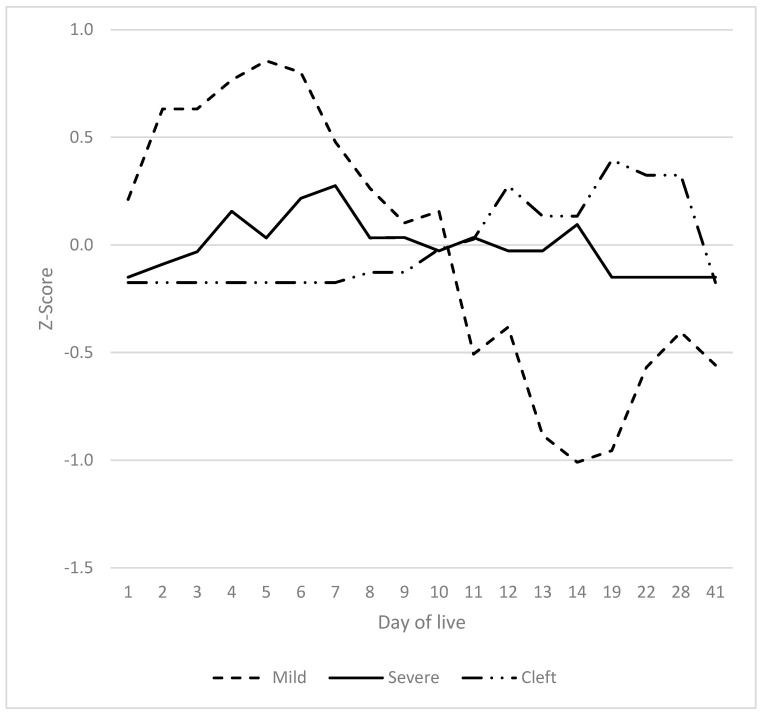
Progression of inflammatory signs in the coronary bands. Scores of fore and hind limbs were added together before the Z-transformation.

The initial indications of inflammation of the coronary bands manifested by swelling and redness were evident by postnatal day 2, reaching a peak around days 5 to 6 (Figure 9). The values obtained on days 2 to 5 were found to be significantly higher than those recorded on days 1 and 8 to 41. The manifestation of more severe signs, characterised by exudation and crusting, occurred with a delay of approximately one day. However, the prevalence of this phenomenon was low, with only a small number of animals affected. A substantial increase in values was observed on day 7 in comparison to days 1 to 3, 10, 12, 13, and 19 to 41. From day 11 to day 26, a significant enhancement in the health of the coronary bands was observed. Concurrently, the presence of cracks in the claw wall was observed from day 12 to day 26. The values obtained on the first day were found to be significantly lower than those recorded between days 12 and 26, and the values obtained on days 12, 19 to 26 and 41 were found to be significantly higher than those recorded on days 1 to 11. Claw swelling was observed from the 2nd week of life.

### 3.7. Summarised Time Patterns

**Figure 10:**
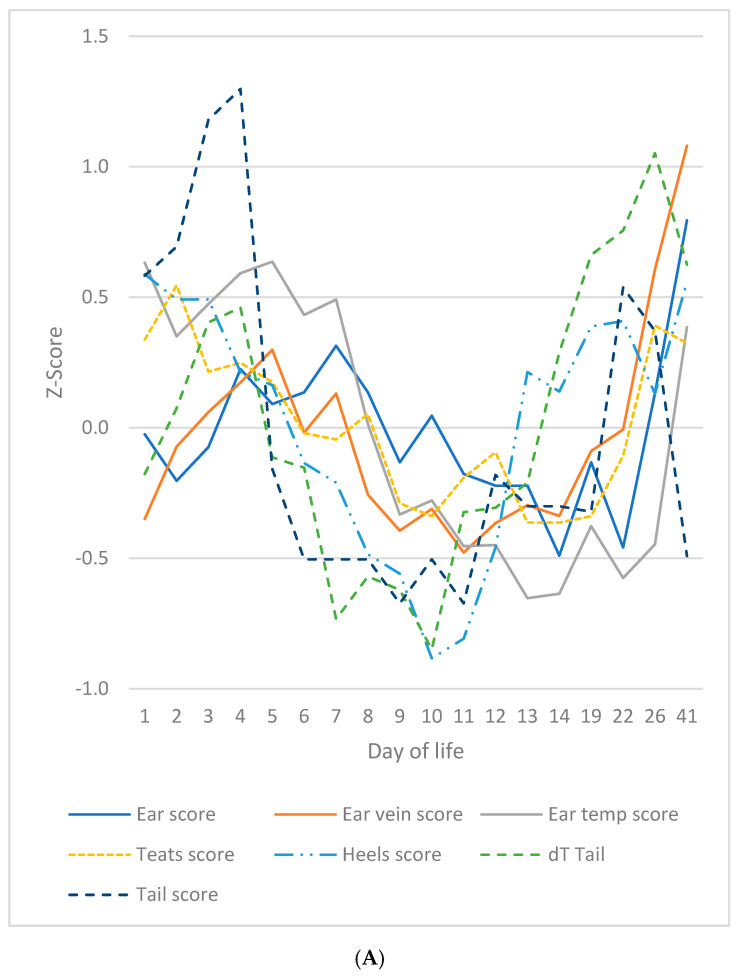

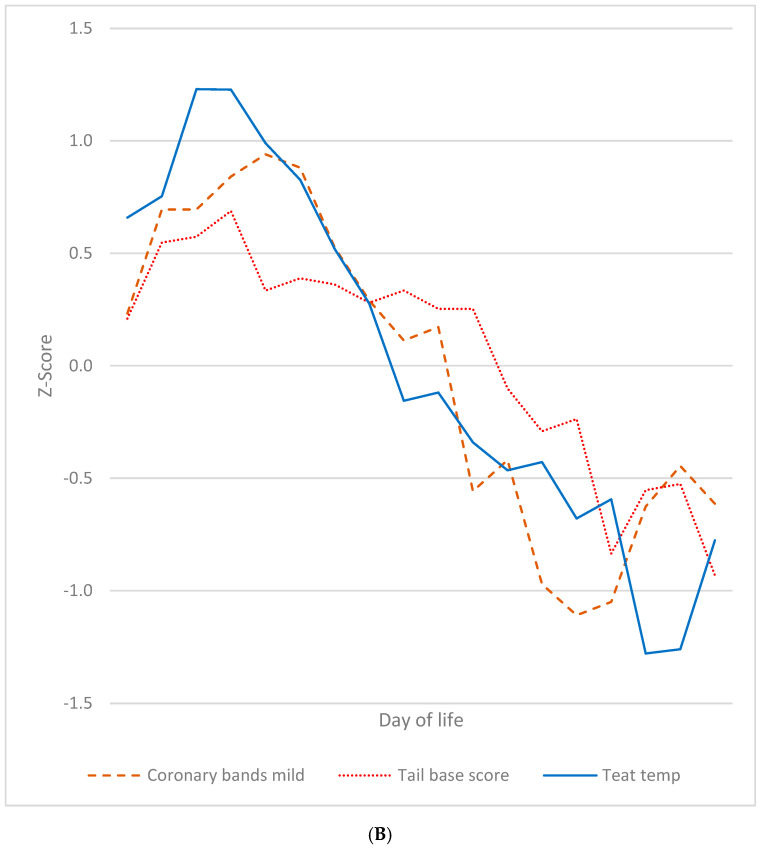
Summary of the progression of scores for the tail, ears, teats and heels, together with ear temperature and the temperature reduction from the tail base to the tail tip (**A**) as compared to the progression of scores for swelling and redness in coronary bands, the total tail base score and teat temperature (**B**). All data are Z-transformed; dt Tail: loss of temperature from tail base to tail tip.

In summary, two main patterns emerged during the study period. A considerable proportion of the characteristics under scrutiny manifested a bimodal distribution, characterised by two pronounced elevated peaks around days 4 and 22/23 relative to day 1, and the time span between days 8 and 12. This pattern manifested itself most distinctly in the SINS signs of the ears, teats, heels, and tail, as well as in the temperature patterns of the ears and tail (Figure 10A). An additional group of traits exhibited a substantial increase around day 4, subsequently followed by a significant decline after the midpoint of the study period, reaching a minimum around days 22 to 41 (Figure 10B). Examples of this pattern include mild inflammation of the coronary band, tail base score and teat temperature. The course demonstrated a high level of significance, with a strong correlation observed between tail base vs. coronary bands and teat temperature vs. coronary bands (r = 0.840; *p* < 0.05), as well as between teat temperature vs. tail base (r = 0.841; *p* < 0.05). The correlations between all parameters of Figure 10A ranged from 0.44 to 0.86 (mean: 0.65; *p* < 0.05).

### 3.8. Chronological Sequence of SINS Characteristics

**Figure 11:**
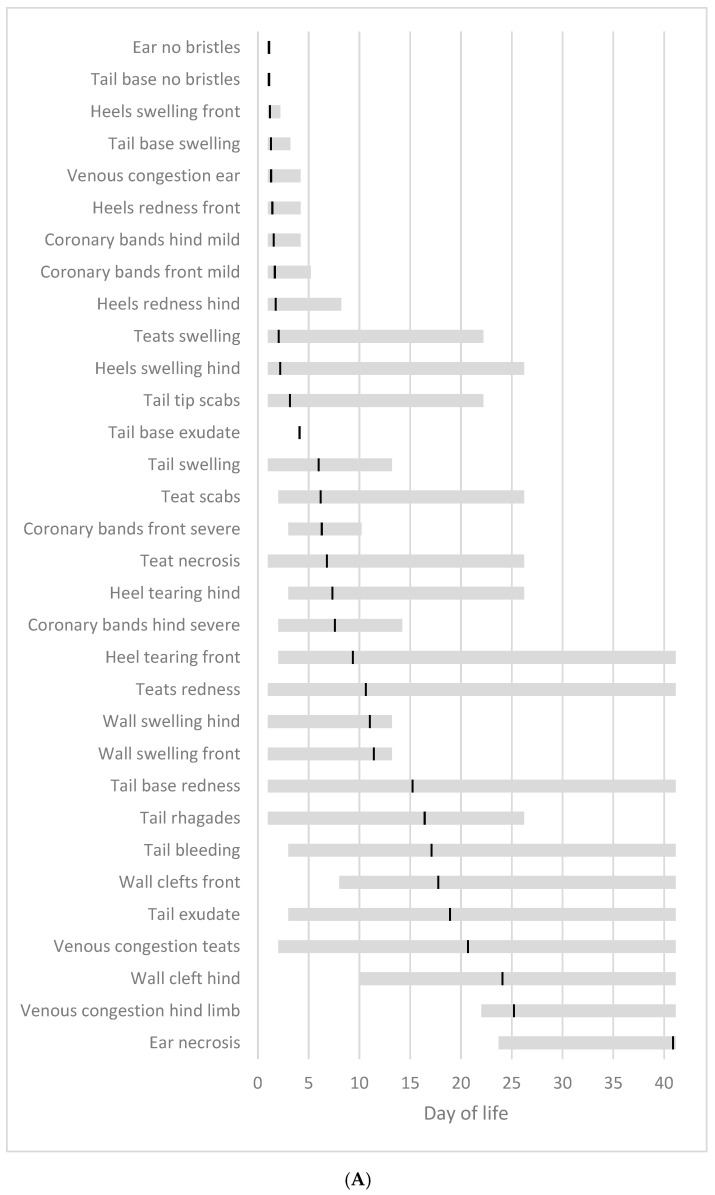

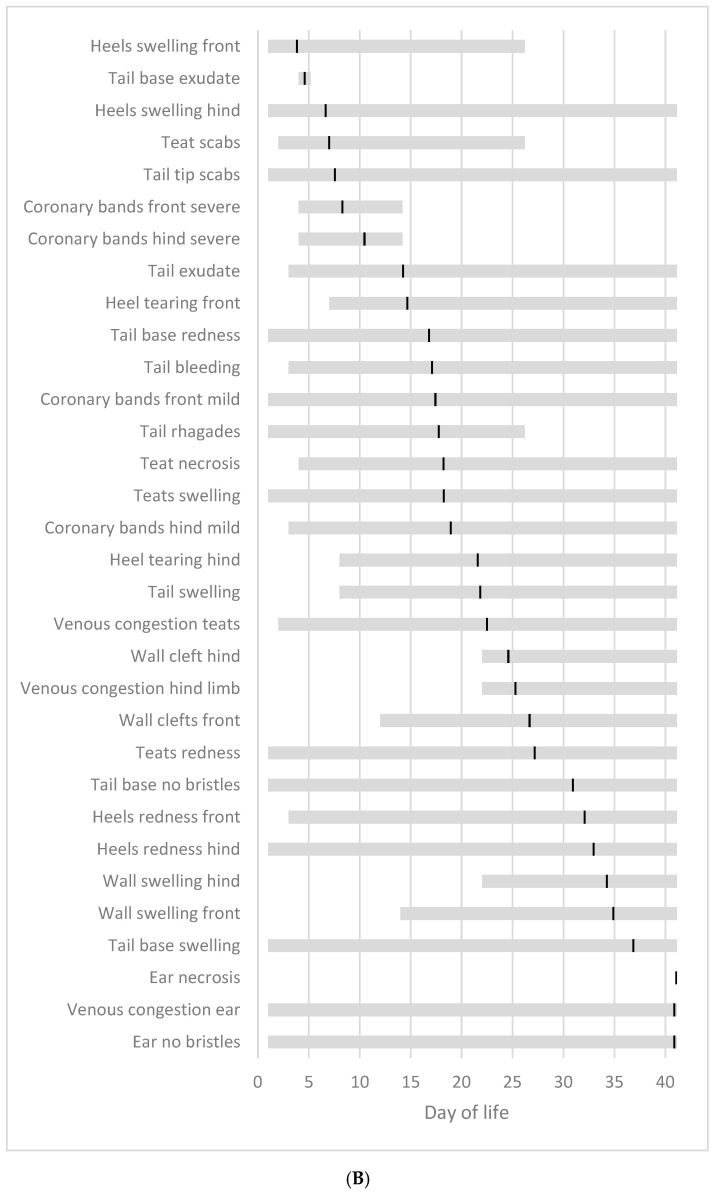
Times of first (**A**) and last (**B**) occurrence of SINS signs in individual piglets. The grey bars show the time interval for the first appearance (**A**), and the time interval for the latest visibility (**B**), respectively, during the 41-day observation period. The black line shows the time for the mean occurrence (**A**) and for the mean latest observation (**B**). The data are sorted according to the means.

It was evident that a significant number of SINS signs manifested from the first day of observation or within the initial few days (Figure 11A). The temporal sequence of the initial appearance of each SINS sign within the study is delineated by the grey bar. It was observed that all piglets exhibited a loss of bristles at the ears and tail base from the study’s inception. Conversely, the onset of ear necrosis did not occur before day 24, and this sign was only observed on the final day of the study period. The black bar denotes the mean time at which the sign first appeared. As demonstrated in Figure 11B, the temporal period during which a SINS sign was last observed in the piglets is indicated, along with the mean time point at which the sign was last observed (black bar). A considerable proportion of these signs did not dissipate until the conclusion of the study period on day 41.

**Figure 12:**
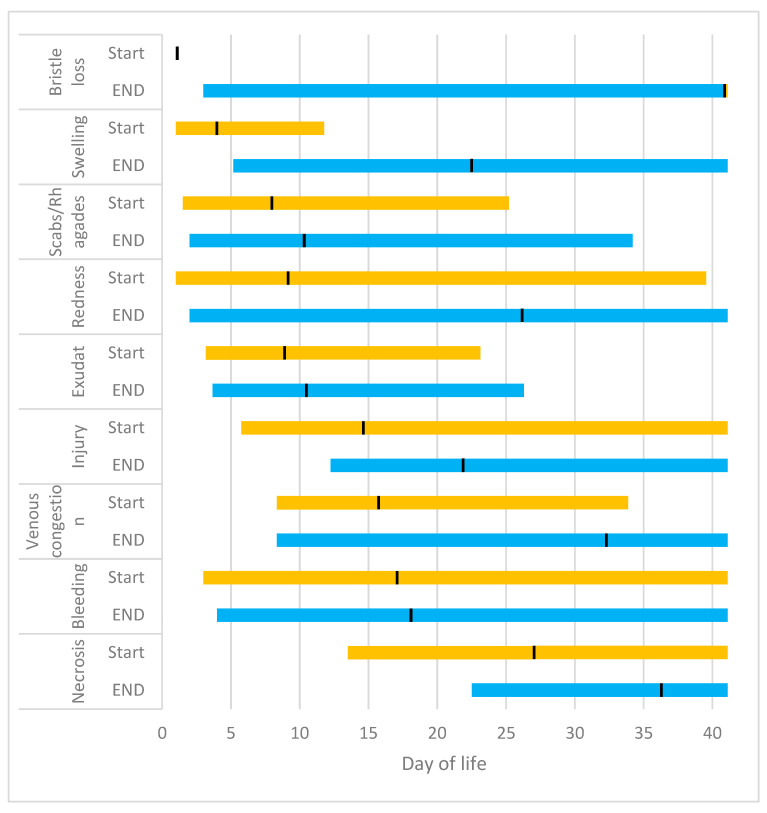
The start and end times of the visibility of SINS grades, independent of the body part.

Irrespective of the body part, the initial signs of the disease were characterised by bristle loss, which occurred on average from day 1. Subsequently, an increase in size was observed, which manifested on average from day 3 onwards. Subsequently, scabs/ragades emerged from day 7, and redness and exudate from day 8. By the fifteenth day, injuries had become apparent, and by the sixteenth day, vein congestion was observed. By day 17, bleeding was evident, and by day 27, necrosis was seen (Figure 12). The loss of bristles remained moderate until the conclusion of the study. The signs of necrosis were no longer visible on day 36; the vein congestion on day 32; the redness on day 26; the swelling on day 23; the signs of injury on day 22, and the bleeding on day 18. The remaining signs disappeared on average before day 11. However, in the majority of cases, signs of SINS, irrespective of the affected body part, were still observable in some piglets until the end of the study.

**Figure 13:**
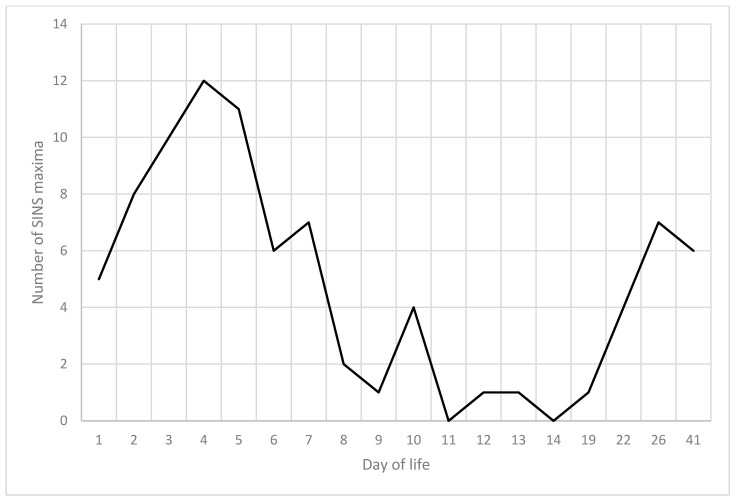
SINS maxima per individual piglet by day of life.

The 59 piglets under scrutiny in the present study demonstrated a total of 86 maximum SINS values (Figure 13). It was observed that five of the piglets had already attained their maximum SINS value by the first day of life. Nevertheless, the preponderance of these events transpired within the timeframe spanning from days 3 to 5. A paucity of peaks was observed between days 11 and 19, with some piglets not reaching their maximum values until the third week of life. In 33% of the piglets, two peaks were clearly visible in the SINS score. In 48% of piglets, peaks were only visible in the first week; in 9% of piglets, peaks were only visible in the fourth week and in a further 9% of piglets, no clear peak was visible in this longitudinal study.

### 3.9. Association Between Individual SINS Scores at Different Days of Life

The repeatability of the SINS score is expressed as the coefficient of determination for the correlation between the SINS scores of all piglets on different examination days (Table 5). The SINS score on day 1 did not permit conclusions to be drawn about subsequent days. The SINS scores of the piglets on days 3 to 5 demonstrated a 40% agreement rate. As was the case in the preceding period, a similar set of values was identified for the period between days 7 and 11, with agreement rates ranging from 40% to 60%. These days were characterised by the lowest SINS levels throughout the study period. Subsequent to this, there was a substantial decline in agreement, attributable to the manifestation of signs in the piglets at varying temporal points.

## 4. Discussion

### 4.1. Findings Derived from the Longitudinal Design of the Study

SINS is a syndrome in swine that is characterised by varying degrees of inflammation, which can ultimately result in necrosis [4,7]. A number of studies undertaken in different countries have demonstrated that neonates [5], piglets and even finishers are susceptible to SINS with a high degree of prevalence [1,2,3,6,8]. The anatomical regions of the tail base, tail tip, ears, teats, coronary bands, heels and claws can be affected in varying proportions, yet with a high degree of overlap [5,6]. On the basis of practical observations and recommendations, SINS studies have been conducted mostly in two narrowly defined time slots around the fourth day of life and one week after weaning. However, when comparing the two time slots, each study used different individuals during the first and the fourth week of life, while longitudinal studies of individual piglets were lacking.

A number of disparities were identified between suckling piglets and weaners in former studies [7,8]. A proportion of these disparities may be attributable to the comparison of disparate individuals. The consideration of day-of-life effects within the life phase of suckling piglets may also have resulted in a loss of information due to the use of different animals on different days. The rationale behind this phenomenon is likely to be that on a given day of examination, different piglets may be in different stages of inflammation. Early inflammatory mediators such as histamine, bradykinin and prostaglandins have been observed to induce dilatation of the arterioles, and subsequently, the postcapillary venules. This results in redness and warmth, thereby ensuring that a greater number of defence cells, immune cells and defence factors reach the site of the action [14]. Subsequent to this, endothelial cells undergo activation, a process which results in the release of procoagulants. These, in turn, express tissue factors that initiate the process of coagulation. The inflammatory process, thus, enhances coagulation through the action of cytokines such as IL-1, TNF-α, TF and thrombin. Furthermore, thrombin and thrombin-activated cells have been shown to promote inflammation [15]. It is evident that the initiation of an inflammatory response is not feasible in the context of practical field studies. Consequently, the variability observed in the results is likely attributable to the heterogeneity of the inflammatory responses among different animals starting at day one of the present study. This correlation can, therefore, also be assumed for practical conditions.

The present results confirm the variability and progression of SINS, with a peak during the first week of life and a second peak during the fourth week of life. The reappearance of typical SINS signs well in advance of the commencement of the weaning process on day 28, thus initiating an earlier second peak, suggests that weaning itself cannot be responsible for the occurrence of the second peak. Nevertheless, the process of weaning could have been a contributing factor to the development of the second peak. For the start of the second peak, it is more probable that the aetiology of the condition is associated with metabolic overload [16,17,18].

The present study also demonstrates a high variability of individual results with dispersion around the respective peak. This indicates that when piglets are compared on days 1, 4 and 8 of life, the total variance of the SINS scores increases and the assignment of target effects (e.g., boar genetics or husbandry measures) becomes more difficult. From a pragmatic standpoint, it is imperative to curtail the age of the piglets examined to the greatest extent feasible, whilst incorporating the day of life into the analysis process. The hypothesis that the SINS scores of suckling piglets can be found again in weaners has not been confirmed. In particular, during the periods of heightened activity of the two peaks, a lack of correlation was observed between the SINS characteristics of the piglets after a mere 2 to 3 days.

Consequently, it is not feasible to predict the future performance of individual animals based solely on their historical performance, even in cases where the group data precisely replicates the double-peak curve. Research has demonstrated that the quality of the sow exerts a significant influence on the SINS status of her litter, with this influence persisting until the piglets reach the finisher phase [8]. However, individual animals appear to be even more strongly affected by direct effects [8,19]. It is also important to acknowledge that the frequency of individual animal examinations, despite the minimal stress caused by the individual examination, may have had an influence on the immediate development of SINS signs. This phenomenon could be a contributing factor to the observed low levels of repeatability. Conversely, this paradigm offers a promising intervention strategy, as the variability of the SINS characteristic in individuals can be modulated based on situational demands. Consequently, countermeasures should facilitate expeditious enhancement. This assertion is corroborated by the findings of Reiner et al. [8]. Moreover, the implementation of basic husbandry practices, namely the provision of mycotoxin-tested hay and the administration of hygienised water via troughs, was found to result in a substantial reduction in SINS, from suckling piglets to finishers.

### 4.2. SINS in Young Individual Piglets Can Have One or Two Peaks at Two Distinct Time Points

The results of the study confirm the suspected dual-peak course of the disease, based on the prevalence and distribution of the individual signs of inflammation. The majority of the traits under scrutiny exhibited two peaks, with the first and third weeks demonstrating the most significant results. Nonetheless, discrepancies were observed, with certain characteristics manifesting solely during the initial or secondary peaks. It has been observed that certain traits, including the tail base score, teat scabs, heel swelling and coronary band scores, reached their maximum levels during the first week of life. In contrast, teat redness and wall cracks did not manifest until the third week.

An examination of the maximum overall SINS scores attained by individual piglets further corroborates the dual-peak course of the disease. Although it is highly probable that SINS can be triggered prior to birth [5], this significant finding of the present study led to the confirmation of the hypothesis that the factors triggering the endogenous development of SINS are also and particularly active during the first week of life and from the third week of life onwards. The presented time interval is characterised by the occurrence of adaptation and adjustment processes in piglets [16,17,18]. The time course of SINS symptoms during the initial week of life is directly proportional to the initial surge in metabolic turnover, which occurs in the first days of life. This is followed by a subsequent decline until the tenth day of life [20]. This study demonstrates that metabolic requirements per kilogram of body weight are particularly elevated during the initial week of life. A further challenge is posed by the weight range between 10 and 35 kg, wherein the highest metabolic activity, particularly in the liver and intestines, is concomitant with an immune system that has not yet reached full development [16,17,18]. Although the highest growth performance is achieved in the 75 to 90 kg body weight range, currently, with average daily gains of over 1200 g/animal in certain lines [21], this is significantly less per kg body weight than in younger piglets. This metabolic overload has its correspondence in a significant alteration in the gene expression of the liver in animals affected by SINS in comparison to those not affected by the condition [11,12]. This process entails a shift in metabolic pathways from anabolic to inflammatory and acute phase, as evidenced by the upregulation of pivotal enzymes and mediators [10,11]. The most critical factors for piglet health are water supply and quality, feed composition, ambient temperature, and animal health. These factors are considered to be efficient in triggering SINS [4].

### 4.3. Hypothetical Link Between Endogenous Imbalances and SINS Peaks

The hypothesis is that the initial site of impairment is an overload of the gut and liver. Excessive bacterial proliferation or shifts in the gut microbiome have been demonstrated to result in elevated levels of microbe-associated molecular patterns (MAMPs) within the gastrointestinal tract [22]. The multifactorial aetiology of this condition has been well-documented. In swine, sudden alterations to dietary and environmental factors during weaning have been demonstrated to induce shifts in the microbial composition of the gut, as evidenced by a decline in microbial diversity and the subsequent overgrowth of opportunistic pathogens, such as Escherichia coli [23]. Antibiotic treatment has been shown to cause a rapid and profound shift in the microbial composition, favouring resistant bacteria and reducing commensal populations [24]. Imbalances in dietary fibre, dietary fatty acids and dietary electrolyte balance have been demonstrated to reduce barrier function and enhance the growth of pathogenic bacteria [25,26,27,28]. It is important to note that other potential causative factors may include intestinal diseases, the administration of antibiotics, a temporary lack of food or water, intestinal disease and any cause of coprostasis [29].

In the present study, there were neither sudden alterations in dietary or environmental factors at the time when the second peak of SINS signs started, nor antibiotic treatment or vaccination. However, it cannot be excluded that the overall presentation of food, water, fibre and housing conditions could have been involved in the rise of SINS signs because of the relative endogenous state of the piglets with the increasing performance at in parallel with the second SINS peak [16,17,18].

Such stress factors are likely to exert influence during transitional periods, such as the first days of life and during weaning, and may be responsible for the observed SINS prevalence at these times. In accordance with the tenets of the colonisation resistance concept, the microbiota has been demonstrated to mediate resistance to infection by means of stimulating the innate immune response [30]. However, perturbed interactions have emerged as drivers of various chronic disease states [22] and can trigger pathophysiologies at distant sites and manifest as distinct symptoms. As demonstrated in the research conducted by [31], one example in humans and mice is non-alcoholic fatty liver disease (NAFLD), where a disturbance of the gut microbiome leads to liver inflammation, with some outcomes similar to those described for SINS [4]. Another illustration of this phenomenon in human subjects is inflammatory bowel disease [32], a collective designation for chronic inflammatory diseases, including Crohn’s disease and ulcerative colitis. In addition to a direct shift in the gut microbiome, leaky gut has been demonstrated to play a pivotal role in the pathogenesis of these diseases, a concept that has been extensively elaborated in the field of human medicine [33,34,35].

The extant literature suggests that the maintenance of a stable intestinal barrier is imperative in order to prevent various potentially harmful substances and pathogens from entering the circulation. The term ‘leaky gut’ is used to describe a disruption of the intestinal barrier, which appears to be characterised by the release of bacterial metabolites and endotoxins, such as lipopolysaccharides (LPS), into the bloodstream. This condition, which in humans is primarily triggered by bacterial infections, oxidative stress, a high-fat diet, alcohol consumption or chronic allergens and dysbiosis [36], appears to be closely linked to the development and/or progression of various metabolic and autoimmune systemic diseases, including obesity, non-alcoholic fatty liver disease (NAFLD), neurodegeneration, cardiovascular disease, inflammatory bowel disease and type 1 diabetes mellitus (T1D) in humans [36]. LPS has been demonstrated to play a pivotal role in this process. It has also been identified as a key contributing factor in the initiation and progression of low-grade systemic inflammation [37,38], including in pigs as an animal model [39].

The entry of LPS into portal circulation through a disrupted intestinal barrier has been shown to induce inflammation and metabolic endotoxemia in pigs [40,41,42]. These processes have even been linked to the development of long-term behavioural abnormalities, including tail biting [43,44]. The blood-gut barrier in pigs is a complex, oxygen-dependent structure that is susceptible to disruption. As demonstrated by Pearce et al. [45,46,47], inadequate water supply and impaired thermoregulation can result in diminished intestinal perfusion, leading to the dissolution of the intestinal surface and crypts. This phenomenon is accompanied by a loss of mucosal protection. In addition, the expression and function of tight junctions are disrupted. This has been shown to result in the modulation of the gut microbiome, with altered microbial metabolites (MAMPS, LPS) inducing oxidative stress and inflammation [48,49]. Inadequate fibre intake in pigs exacerbates this condition, as in the absence of short-chain fatty acids, the gut microbiome metabolises gut mucosal glycoproteins and thus the first layer of the blood-gut barrier [50,51,52,53].

Increased passage of MAMPs and gut bacteria into the bloodstream due to disruption of the blood-gut barrier has been shown to promote inflammation of the gut, liver and systemically, as well as the development of metabolic syndrome in swine [50,54,55,56]. In sows, an increase in inflammatory markers such as ROS, IL-6 and TNFa has been observed as early as mid-pregnancy [50]. These markers have been linked to various adverse outcomes, including coprostasis, fetal developmental stagnation, increased embryonic losses and decreased neonatal vitality [57,58,59,60,61,62]. Finally, the intestinal health of sows and piglets is impaired [63,64]. The aforementioned effects constitute the foundation of the hypothesis of SINS pathogenesis [4], a hypothesis which has been substantiated on numerous levels by clinical chemistry [9], pathology [5,8], metabolomics [9] and transcriptomics [10,11]. Significant evidence for the hypothesis is also provided by the identification of candidate genes for SINS pathogenesis, based on comparisons of pigs with significantly different SINS susceptibility [11,65,66].

Liver inflammation in suckling piglets with SINS, accompanied by severe metabolic disturbances, has been demonstrated by transcriptomic and metabolomic studies [9,10,11]. This finding may indicate a potential link between a state of metabolic tension and the onset of SINS, as evidenced in the present study. Furthermore, overloading the liver can result in coagulation disorders in pigs with spontaneous bleeding, for example, from the tail [67]. Furthermore, research has indicated an association between blood coagulation disorders, such as elevated fibrinogen levels and thrombocytopenia, and SINS [9]. This phenomenon elucidates the spontaneous haemorrhages observed in the tail region during SINS peaks and as early as day 12 of life. In such cases, the hypothesis of biting could be safely excluded on the basis of daily observation of the animals and the absence of bite marks.

The presence of the inflammatory mediators that are released in response to MAMPs has been identified in SINS-positive piglets through the utilisation of clinical chemistry, metabolomics and transcriptomics methodologies (see [9,10,11]). It has been established that the activation of vascular endothelium and lymphocytes is a key process in this pathway. An increase in the production of acute phase proteins is also observed, as is the influx of immunoglobulins, complement and immune cells, monocytes and granulocytes from the blood into the tissue. This process ultimately results in thrombosis of small blood vessels [68]. The histopathological demonstration of these processes has been reported in SINS [5,8]. The absence of clinical evidence of bleeding in the present study indicates that the highest SINS grades did not occur here.

Mycotoxins such as deoxynivalenone (DON) have also been demonstrated to exert a deleterious effect on the integrity of the blood-intestinal barrier. The inhibition of protein biosynthesis has been demonstrated to result in a subsequent degradation of tight junctions [69]. This has been demonstrated to result in the inflammation of the intestine and liver in pigs [69,70]. Mycotoxins have been demonstrated to act synergistically with LPS [71,72,73,74]. It has been demonstrated that both substances promote mutual absorption in the gut and inhibit mutual degradation in the liver and overall hepatic metabolism [75,76]. LPS and mycotoxins have been detected in the milk of sows and are believed to be the causative agents of necrosis on the tails, ears and coronary bands of suckling piglets [77,78,79,80]. LPS and other MAMPs have been shown to originate from physiologically present gut bacteria [74,81] and are continuously detoxified by the liver and bile [75,82]. There were no obvious problems with mycotoxins in the present study.

The facts and hypotheses presented on the causes of SINS provide significant foundational insights into the clinical observations documented in this study, despite the absence of investigation and elucidation of the specific factors underlying these findings, a subject that merits attention in future research endeavours.

### 4.4. SINS Characteristics and Body Temperature

The unfavourable influences on piglets during their first week of life and around weaning not only result in the typical clinical signs of SINS. Moreover, these effects are also observed in the temperature curves at the tail, ears and teats. It was observed that the temperature of the ears increased at both SINS peaks. Research has demonstrated a strong correlation between ear temperature and body temperature (r = 0.85), thus suggesting its potential as a reliable indicator of core temperature in pigs [83]. Concurrently, the significance of pigs’ ears as thermoregulatory organs, facilitating the dissipation of excess heat, has been well-documented [84]. Consequently, an elevated ear temperature during the SINS peaks is indicative of an augmented body temperature and a heightened requirement for thermal regulation. This correlation is consistent with the widely recognised importance of thermoregulation for intestinal stability [45,46,47].

The present longitudinal study confirms the parallelism between ear temperature and the occurrence of the most important signs of SINS on the ears, tail, teats and heels. Furthermore, a high degree of parallelism is evident in the temperature curves at the base of the tail. However, a significant increase in the temperature decline from the base of the tail to the tip of the tail is observed during both SINS and temperature peaks.

As the temperature from the base of the tail to the tip exhibited a particularly sharp decline at the two times when the SINS signs in different body parts were most pronounced, it can be interpreted that the drop in temperature is indicative of impaired blood flow in the tail due to inflammation at the tail base. Exact measurements of the blood flow rate in the tail were not conducted; however, it is generally accepted that circulatory disturbances are manifested by a drop in temperature [84,85]. It is also worthy of note that the decline in temperature during the initial SINS peaks was concomitant with a substantial augmentation in tail temperature at the base, in a region that did not yet exhibit any clinical abnormalities. Such circulatory disturbances can be caused by changes in blood and plasma composition, but also by smooth muscle spasm or inflammation [86]. It has been established that SINS signs in the tail are associated with circulatory disturbances due to vascular inflammation, including intimal proliferation and partial or complete thrombosis of the vessels at the base of the tail [5,8]. The present study demonstrates a striking parallelism between the additive tail score and the temperature drop from the tail base to the tail tip.

The teats presented a particular situation. As with the tail and ears, a gradual decrease in teat temperature was observed over the initial part of the study period. An increase in teat temperature was observed to coincide with the initial SINS peak, accompanied by a concurrent increase in ear and tail base temperature. In contrast, a decline in tail temperature was observed coinciding with the subsequent SINS peak, accompanied by a decrease in ear temperature. Another noteworthy observation pertained to the distinct impact of sex, a phenomenon previously documented by Konders-van Gog et al. [1]. It was observed that there was a notable similarity in the temperature curves of male and female piglets. Initially, both exhibited an increase in temperature (day 4), subsequently followed by a decrease in temperature (3rd week of life). The significantly more pronounced teat signs of female piglets under SINS, especially in terms of swelling, scab formation and necrosis, suggest that female sex hormones may play a modifying role at a given SINS level and floor. As demonstrated in the relevant literature [87], female piglets have been shown to be more susceptible to alterations of the teats than male piglets. It is evident that the extent of alterations in the teats is contingent on the nature of the flooring material employed. Nevertheless, the extant evidence would appear to suggest that SINS is also significantly implicated in the development of teat inflammation and necrosis. This assertion is substantiated by substantial sex disparities, pronounced teat alterations in newborn piglets [5], discernible breed variations, and the impact of boars on teat alterations [6]. Notably, the heritability for teat alterations (0.33; [2]), while constant, substantiates the validity of these observations. The present study demonstrated a strong correlation between the teat score and the scores of the other body parts and the total SINS score, thus emphasising the significance of SINS in the manifestation of teat alterations in piglets. However, further studies are required to elucidate the underlying factors and mechanisms.

A further peculiarity of the teats was that the initial temperature peak was directed upwards, whilst the subsequent temperature peak was directed downwards. As indicated by the results of the present study, on the third to fourth day post-partum, both male and female teats exhibited an elevated temperature. This phenomenon was concomitant with the initial swelling and erythema of the teats and coincided with the onset of scabbing and necrosis. A reversible decrease in temperature was observed at the second peak in teat temperature, which occurred around the third week of life. A comparison of the typical course of inflammation reveals that it is characterised initially by increased blood flow and heat, and later by restricted blood flow to the downstream areas. It can be hypothesised that the two directions may represent expressions of the same inflammatory effect, albeit at different temporal points [88].

Despite the absence of any statistical difference in teat temperature between the sexes, it was observed that females exhibited a significantly higher incidence of teat swelling, scab formation and necrosis. The results of this study indicate that the tissue of female piglets may be more susceptible to a given level of inflammation, which may be attributable to hormonal interactions.

### 4.5. SINS Characteristics at the Claws

The interaction between SINS and the floor, which can be assumed for all tissues close to the ground, also applies to the heels. Notwithstanding, the present study did not aspire to provide a comprehensive characterisation of such interactions. The presence of heel swelling on the first day of life, followed by redness and cracking, could be taken as an indication of a predisposing role of SINS. Nevertheless, there is a paucity of compelling evidence to support this hypothesis. The study by Koenders-van Gog et al. [1] demonstrates a direct correlation between SINS and inflammation in the coronary band and heel area, which is independent of the floor. This effect was particularly pronounced and significant during the first day of life. The direct effect of SINS on the heels then decreased from day to day in favour of the floor, but the overall damage to the heels remained significantly correlated with the SINS score (calculated without claw characteristics) until the end of the study. Consequently, the heightened vulnerability of the heels to damage from the floor due to SINS was documented. In the present study, substantial swelling of the heels was observed from the first day of life, accompanied by redness with a peak around days 2 to 5 and tearing with a peak around day 11. However, the available data did not allow for differentiation between the endogenously induced SINS component and the mechanical stress caused by the floor, and this remains a topic for future studies.

### 4.6. Limitations of the Study

The merits of a longitudinal study are evident in a number of respects (see [89]). For instance, the identification of temporal dynamics, such as peaks or recovery, and the investigation of critical time windows, is facilitated by intra-individual comparisons. This is due to the fact that such comparisons enable the analysis of changes within individuals to be conducted independently of the basic differences that exist among animal groups. The investigation revealed that the archetypal double-peak course of SINS manifests in a mere one-third of piglets, with the remainder arising from the amalgamation of piglets with a primary focus within the initial 52% of the animals and piglets with a focus within the fourth week of life (19%). Furthermore, it is possible to observe the sequence of SINS signs within a shorter period of time and, thus, interpret mild signals as precursors of more severe changes. The sequence of SINS signs anticipated from preliminary studies was subsequently confirmed, albeit only partially discernible in individual animals. This finding demonstrates a limitation of the study in that repeated examination of the animals, despite only low overall stress [90], could lead to an interaction between the examination and the result [91]. In order to assess this effect, it would have been necessary to carry out the examinations with automatic observation and documentation without manual intervention (lifting) of the animals. However, this was not possible in the present study. It is evident that, given each piglet functions as its own control and inter-piglet variance is diminished, such an analysis is less susceptible to interference and possesses greater statistical power, even when utilising smaller samples. Nevertheless, despite the meticulous documentation of characteristics, the number of 59 individuals represents the most significant limitation of the present study. A further limitation arises from the known fact that SINS is significantly influenced by genetics [12,65] of sows [7] and boars [2,6] and by husbandry conditions [8,19]. It should be noted that the present study did not encompass these elements in its design. The objective of the pilot study was to examine the progression and mechanics of SINS in subjects who were longitudinally monitored from the first day of life to day 41 of the weaning period. This investigation was undertaken to ascertain the fundamental principles of the syndrome. The present study could only consider one scenario from a theoretically almost infinite number of possible combinations of different genetics and housing conditions. However, given the observation of SINS signs under the specified conditions, it was possible to investigate the questions posed by the longitudinal study. It is regrettable to report that it was not feasible to continue monitoring the piglets beyond day 41. Nevertheless, the time span was sufficient to indicate the commencement of a decline in SINS signs for the second peak. Despite the persistence of indications of SINS in finishers [8], the assessment of the SINS event at later time points appears to be of diminished interest. This is attributable to the increasing overlap of endogenous and exogenous effects [4]. The high comparability of the SINS scores with other studies conducted on day 3/4 and in the third week of life [1,2,3,6,8] lends support to the relative general validity of the results. It is recommended that subsequent studies endeavour to identify the individual factors that contribute to the SINS effect in detail in targeted trials. The purpose of this would be to support livestock farmers and veterinarians in their decision-making process to identify the most important factors on a case-by-case basis. Evidence from both scientific research [8] and practical experience indicates that such measures have the potential to substantially mitigate the SINS problem.

## 5. Conclusions

The present study corroborates the two-peak configuration of SINS, with a peak observed in the first and fourth weeks of life. The individual contributions of individual animals to the peaks can vary greatly. Consequently, it is not feasible to predict the subsequent course of SINS based on individual animals. In the context of practical investigations and studies, it is imperative to consider the pronounced influences of the day of life. This can be achieved by establishing a clearly defined investigation time window and by incorporating the day of life into the evaluation process. The period between the first and fourth week of life is not considered suitable for investigating SINS. The transfer of details concerning the study results to other genetic-management combinations is only possible to a limited extent.

## Figures and Tables

**Table 1:** Prevalence of clinical signs at the tail base and tail tip (%).

Day of Life	Tail Base					Tail/Tail Tip						
	No Bristles	Swelling	Redness	Exudation	Necrosis	Swelling	Scabs	Rhagades	Exudation	Necrosis	Ring Constriction	Bleeding
1	100.0	83.1	3.4	0.0	0.0	5.1	11.9	1.7	0.0	0.0	0.0	0.0
2	100.0	94.8	13.8	0.0	0.0	3.4	10.3	0.0	0.0	0.0	0.0	0.0
3	98.3	98.3	13.8	0.0	0.0	1.7	6.9	0.0	5.2	0.0	0.0	1.7
4	100.0	100.0	14.3	3.6	0.0	1.8	3.6	0.0	1.8	0.0	0.0	1.8
5	92.9	100.0	0.0	1.8	0.0	33.9	0.0	0.0	1.8	0.0	0.0	0.0
6	94.6	100.0	3.6	0.0	0.0	55.4	0.0	0.0	0.0	0.0	0.0	0.0
7	92.9	100.0	3.6	0.0	0.0	53.6	0.0	0.0	0.0	0.0	0.0	0.0
8	89.3	98.2	3.6	0.0	0.0	66.1	0.0	0.0	0.0	0.0	0.0	0.0
9	94.6	100.0	0.0	0.0	0.0	60.7	0.0	0.0	0.0	0.0	0.0	0.0
10	85.7	100.0	3.6	0.0	0.0	71.4	0.0	0.0	0.0	0.0	0.0	0.0
11	91.1	98.2	0.0	0.0	0.0	66.1	0.0	0.0	0.0	0.0	0.0	0.0
12	73.2	91.1	1.8	0.0	0.0	50.0	0.0	0.0	0.0	0.0	0.0	3.6
13	64.3	85.7	3.6	0.0	0.0	62.5	0.0	0.0	0.0	0.0	0.0	1.8
14	71.4	82.1	3.6	0.0	0.0	55.4	0.0	0.0	0.0	0.0	0.0	1.8
19	33.9	80.4	3.6	0.0	0.0	19.6	0.0	0.0	1.8	0.0	0.0	0.0
22	45.5	89.1	1.8	0.0	0.0	18.2	3.6	1.8	3.6	0.0	0.0	1.8
26	56.4	80.0	1.8	0.0	0.0	1.8	0.0	3.6	3.6	0.0	0.0	0.0
41	38.5	69.2	3.8	0.0	0.0	0.0	0.0	0.0	0.0	0.0	0.0	0.0

**Table 2:** Prevalence of clinical signs at the ears and teats (%).

Day of Life	Ears			Teats				
	No Bristles	Venous Congestion	Necrosis	Venous Congestion	Swelling	Redness	Scabs	Necrosis
1	100.0	86.4	0.0	0.0	40.7	32.2	0.0	5.1
2	100.0	79.3	0.0	5.2	39.7	29.3	1.7	17.2
3	100.0	84.5	0.0	1.7	34.5	6.9	6.9	19
4	100.0	96.4	0.0	1.8	33.9	3.6	10.7	21.4
5	100.0	91.1	0.0	1.8	30.4	7.1	7.1	19.6
6	100.0	92.9	0.0	1.8	26.8	7.1	0.0	16.1
7	100.0	100.0	0.0	1.8	23.2	7.1	1.8	16.1
8	100.0	92.9	0.0	0.0	26.8	16.1	1.8	12.5
9	100.0	82.1	0.0	0.0	12.5	8.9	0.0	10.7
10	100.0	89.3	0.0	0.0	8.9	5.4	1.8	12.5
11	100.0	80.4	0.0	0.0	12.5	17.9	0.0	8.9
12	96.4	82.1	0.0	0.0	14.3	21.4	0.0	10.7
13	100.0	78.6	0.0	0.0	12.5	8.9	0.0	5.4
14	100.0	67.9	0.0	0.0	8.9	14.3	0.0	3.6
19	98.2	83.9	0.0	1.8	8.9	14.3	0.0	3.6
22	100.0	69.1	0.0	0.0	9.1	29.1	0.0	7.3
26	100.0	90.9	1.8	1.8	7.3	63.6	1.8	7.3
41	100.0	88.5	30.8	7.7	23.1	46.2	0.0	0.0

**Table 3:** Prevalence of clinical signs at the coronary bands, heels, claws and hind limb (%).

Day of Life	Coronary Bands						Heels						Claw Wall		Hind Limb
	Swelling/Redness		Exudation		Cleft		Swelling		Redness		Tearing				Venous Congestion
	Front	Hind	Front	Hind	Front	Hind	Front	Hind	Front	Hind	Front	Hind	Front	Hind	
1	62.7	67.8	0.0	0.0	0.0	0.0	30.5	39.0	69.5	59.3	0.0	0.0	1.7	1.7	0.0
2	84.5	87.9	0.0	1.7	0.0	0.0	6.9	10.3	91.4	82.8	1.7	0.0	5.2	8.6	0.0
3	87.9	84.5	1.7	1.7	0.0	0.0	1.7	10.3	96.6	82.8	0.0	1.7	0.0	3.4	0.0
4	94.6	91.1	3.6	5.4	0.0	0.0	0.0	7.1	89.3	75.0	10.7	12.5	0.0	0.0	0.0
5	96.4	98.2	3.6	1.8	0.0	0.0	0.0	3.6	91.1	73.2	3.6	16.1	0.0	0.0	0.0
6	94.6	94.6	5.4	5.4	0.0	0.0	0.0	5.4	85.7	55.4	7.1	26.8	0.0	0.0	0.0
7	76.8	80.4	8.9	3.6	0.0	0.0	0.0	3.6	80.4	57.1	8.9	26.8	0.0	0.0	0.0
8	71.4	64.3	3.6	1.8	1.8	0.0	0.0	3.6	67.9	50.0	12.5	32.1	0.0	0.0	0.0
9	58.9	60.7	1.8	3.6	1.8	0.0	0.0	5.4	64.3	46.4	14.3	30.4	1.8	7.1	0.0
10	64.3	60.7	1.8	1.8	3.6	1.8	0.0	5.4	48.2	39.3	35.7	37.5	7.1	8.9	0.0
11	26.8	32.1	1.8	3.6	5.4	1.8	0.0	5.4	48.2	44.6	48.2	39.3	17.9	17.9	0.0
12	35.7	35.7	1.8	1.8	12.5	3.6	0.0	1.8	62.5	58.9	37.5	37.5	53.6	44.6	0.0
13	10.7	10.7	1.8	1.8	7.1	3.6	0.0	5.4	91.1	75.0	8.9	14.3	100.0	100.0	0.0
14	8.9	0.0	3.6	3.6	7.1	3.6	3.6	8.9	87.5	66.1	7.1	17.9	100.0	100.0	0.0
19	7.1	7.1	0.0	0.0	12.5	7.1	1.8	1.8	96.4	83.9	1.8	8.9	87.5	98.2	1.8
22	25.5	27.3	0.0	0.0	7.3	9.1	0.0	1.8	96.4	87.3	3.6	7.3	78.2	94.5	25.5
26	32.7	36.4	0.0	0.0	7.3	9.1	3.6	7.3	90.9	63.6	3.6	20.0	61.8	70.9	36.4
41	30.8	23.1	0.0	0.0	0.0	0.0	0.0	3.8	96.2	96.2	3.8	0.0	96.2	100.0	0.0

**Table 4:** Temperatures at the tail base, tail tip, ears and teats (mean ± SD, °C).

Day of Life	Tail Base (°C)	Tail Tip (°C)	Delta T (°C)	Ears (°C)	Teats (°C)
1	35.7 ± 0.9	31.5 ± 2.4	4.2	31.5 ± 1.8	36.7 ± 1.2
2	35.8 ± 1.4	31 ± 2.6	4.9	31.6 ± 1.8	37.0 ± 1.2
3	36.3 ± 1.4	30.5 ± 3.1	5.8	32.6 ± 1.8	37.6 ± 0.6
4	36.6 ± 0.8	30.6 ± 2.3	5.9	33.0 ± 1.9	37.5 ± 0.6
5	36.5 ± 0.6	32.1 ± 2.3	4.4	33.0 ± 2.0	37.3 ± 0.6
6	36.3 ± 0.8	32.1 ± 2.3	4.3	32.2 ± 2.2	37.1 ± 0.6
7	36.3 ± 0.8	33.7 ± 2.6	2.7	32.3 ± 2.8	36.8 ± 0.5
8	35.5 ± 1.3	32.4 ± 3.4	3.1	30.3 ± 3.9	36.6 ± 0.8
9	35.0 ± 1.0	32.1 ± 2.7	3.0	28.7 ± 2.9	36.1 ± 0.7
10	35.2 ± 1.0	32.8 ± 2.9	2.3	28.2 ± 3.4	36.0 ± 0.8
11	34.6 ± 1.3	30.8 ± 3.9	3.8	27.9 ± 3.4	35.8 ± 0.7
12	34.8 ± 1.2	31.0 ± 3.7	3.8	27.9 ± 3.6	35.8 ± 0.8
13	34.7 ± 1.0	30.6 ± 3.2	4.1	26.7 ± 2.0	35.7 ± 0.7
14	34.3 ± 1.1	28.8 ± 3.2	5.5	26.9 ± 2.0	35.5 ± 0.6
19	34.7 ± 1.2	28.2 ± 3.2	6.5	27.7 ± 3.0	35.6 ± 0.6
22	34.1 ± 1.3	27.3 ± 2.4	6.8	26.9 ± 2.5	34.9 ± 0.7
26	34.6 ± 0.8	27.0 ± 2.7	7.6	27.0 ± 2.4	34.6 ± 0.9
41	35.1 ± 1.0	28.7 ± 2.4	6.4	29.9 ± 3.1	35.3 ± 0.7

SD: Standard deviation; delta T (°C): Temperature drop from tail base to tail tip.

**Table 5:** Repeatability expressed as percentage of explained variance (coefficient of determination) explained by day of life.

Day of Life																		
	1	2	3	4	5	6	7	8	9	10	11	12	13	14	19	22	26	41
1	100.0																	
2	10.7	100.0																
3	16.9	23.4	100.0															
4	12.3	29.1	39.6	100.0														
5	7.9	5.0	24.0	44.3	100.0													
6	0.9	3.0	1.1	10.8	12.4	100.0												
7	2.1	2.2	1.2	8.3	9.3	28.8	100.0											
8	4.1	3.6	4.9	3.3	2.6	14.9	62.2	100.0										
9	5.5	2.3	9.6	0.3	0.1	12.5	43.5	54.0	100.0									
10	10.0	0.5	9.2	0.4	0.9	15.5	46.1	57.5	54.7	100.0								
11	8.6	3.3	4.0	1.8	1.6	16.0	34.0	43.9	39.1	60.2	100.0							
12	7.3	1.5	6.2	2.4	3.0	10.6	45.5	48.3	45.5	58.4	47.4	100.0						
13	0.0	0.2	15.5	0.5	0.9	0.3	7.1	15.5	13.1	23.6	16.9	34.1	100.0					
14	1.5	3.8	2.0	0.1	1.9	6.0	19.9	21.2	13.5	29.5	21.9	26.5	17.2	100.0				
19	8.3	12.8	6.2	7.4	5.1	15.0	17.2	13.0	9.7	14.2	10.6	12.0	13.8	14.3	100.0			
22	9.7	0.1	10.2	0.6	0.1	3.8	17.7	29.2	26.9	34.1	18.5	32.8	26.9	6.2	5.8	100.0		
26	2.9	1.4	6.8	0.7	1.3	5.7	20.1	22.9	38.3	43.7	31.4	48.7	38.6	22.1	11.3	23.6	100.0	
41	0.0	5.1	12.9	0.7	2.3	0.0	2.2	12.0	10.1	1.7	0.4	0.0	0.0	0.0	18.4	3.9	14.6	100.0

## Data Availability

All data are available from the corresponding author by reasonable request.

## Abbreviation

The following abbreviations are used in this manuscript:

SINS	Swine Inflammation and Necrosis Syndrome
